# A Case of Hyperkalemia Versus Pseudohyperkalemia in Chronic Lymphocytic Leukemia

**DOI:** 10.5811/cpcem.2020.3.46481

**Published:** 2020-04-23

**Authors:** Rachel D. Le, Sean P. Geary

**Affiliations:** * Albany Medical Center Hospital, Department of Emergency Medicine, Albany, New York; † Albany Medical Center Hospital, Department of Emergency Medicine and Department of Surgery, Division of Surgical Critical Care, Albany, New York

**Keywords:** pseudohyperkalemia, hyperleukocytosis, chronic lymphocytic leukemia

## Abstract

**Introduction:**

Both hyperkalemia and pseudohyperkalemia occur in the emergency department. True hyperkalemia necessitates emergent treatment while pseudohyperkalemia requires recognition to prevent inappropriate treatment. It is imperative that the emergency physician (EP) have an understanding of the causes and clinical presentations of both phenomena.

**Case Report:**

We present a case of an 88-year-old male with chronic lymphocytic leukemia (CLL) and suspected blast crisis who was found to have elevated serum potassium levels without other manifestations of hyperkalemia and eventually was determined to have pseudohyperkalemia due to white cell fragility.

**Discussion:**

Differentiation of hyperkalemia and pseudohyperkalemia is a critical skill for the EP. We discuss multiple causes of hyperkalemia and pseudohyperkalemia in an effort to broaden the knowledge base.

**Conclusion:**

We present a case of CLL as an unusual cause of pseudohyperkalemia and review common causes of pseudohyperkalemia.

## INTRODUCTION

Hyperkalemia is a potentially life-threatening electrolyte derangement that requires early diagnosis and prompt treatment to prevent significant morbidity and mortality. Pseudohyperkalemia is an in vitro increase in serum potassium without in vivo increase and thus lacks clinical manifestations of hyperkalemia.^1^ Every emergency physician (EP) has encountered pseudohyperkalemia as a result of hemolysis from pre-analytical errors.^2^ Fortunately, the laboratory usually identifies hemolysis at the time of reporting. We present a case of pseudohyperkalemia without apparent hemolysis in a patient with chronic lymphocytic leukemia (CLL), and we present a review of pseudohyperkalemia in the literature.

## CASE REPORT

An 88-year-old male with baseline dementia and known CLL diagnosed in 2016 (although he had not been receiving treatment) initially presented to an outside hospital with bilateral lower extremity edema. There, he was found to have a white blood cell count of 280,000 cells per microliter (cells/μL) (reference range: 4,400–10,400 cells/μL), an increase from 120,000 cells/μL 10 months prior. He was subsequently transferred due to concerns for transformation. At our institution, the patient denied any specific complaints. In discussion with the transferring physician, it was determined that the patient initially presented for lower extremity pain and fatigue, and was found to have negative bilateral deep venous thrombosis studies.

On physical exam, the patient was a cachectic, elderly male found to be normothermic at 36.9º Celsius, with a blood pressure of 114/68 millimeters of mercury, heart rate of 88 beats per minute, respiratory rate of 14 breaths per minute, and oxygen saturation of 95% on room air. Otherwise, he had an unremarkable exam with the exception of symmetric 1+ lower extremity edema without evidence of cellulitis.

Upon arrival, repeat laboratories demonstrated stable hyperleukocytosis of 279,000 cells/μL, but also a potassium of 6.7 microequivalent per liter (mEq/L) (reference range: 3.4–5.2 mEq/L) without reported visible hemolysis. It should be noted the patient had a reported potassium of 4.5 mEq/L at the outside hospital earlier that day. The patient continued to deny any symptoms, and an electrocardiogram (ECG) was obtained that did not show evidence of hyperkalemia. One liter of normal saline and furosemide 20 milligrams were given intravenously with a repeat potassium elevated to 9.4 mEq/L, this time with some hemolysis. Given the rapidly escalating potassium level despite an initial trial of therapy and a normal ECG, a point-of-care potassium was drawn and returned as 3.8 mEq/L. Since this value was more consistent with the outside hospital level and there was a lack of clinical and ECG findings to suggest hyperkalemia, no further interventions were performed in the emergency department.

Over the course of his hospitalization, the patient had multiple elevated potassium levels, usually with interpreted hemolysis, although occasionally without reported hemolysis. In fact, the patient had his apparently elevated potassium treated with a hyperkalemia cocktail of calcium gluconate, insulin, dextrose, and sodium polystyrene on at least one occasion during his hospital stay. The patient never had physical manifestations of hyperkalemia nor were there ECG changes.

## DISCUSSION

In vivo hyperkalemia is a common electrolyte derangement typically seen in chronic kidney disease as well as in acute processes such as rhabdomyolysis and diabetic ketoacidosis. Conversely, pseudohyperkalemia is an in vitro increase in serum potassium. Hemolysis can occur during the pre-analytical process, particularly with mechanical trauma during venipuncture, prolonged tourniquet time, and fist clenching, all of which may cause extracellular movement of potassium from myocytes.^2^

Cases of pseudohyperkalemia associated with thrombocytosis and leukocytosis have been reported in the literature since the 1950s.^1^,^3^ Pseudohyperkalemia has been associated with hyperleukocytosis, more commonly in CLL in adults, but also acute lymphoblastic leukemia in children.^4^–^9^ It is thought that hyperleukocytosis increases cell fragility, making cell lysis more common during specimen collection, particularly with smaller-bore needles, as well as transport and centrifugation. Since leukocytes are more prone to lysis than erythrocytes, laboratory detection may not be apparent if erythrocytes are unaffected. This may lead to the spurious reporting of elevated potassium without the expected caveat of hemolysis and explain why in our case the use of the point-of-care test showed a normal potassium. Moreover, at our institution, we use a pneumatic tube system to transport laboratory specimens, which would further increase the risk of fragile cell lysis compared to a bedside test without transport.

Although we highlight pseudohyperkalemia in the setting of hematologic malignancy, rarer etiologies of pseudohyperkalemia exist in the literature. There are cases of pseudohyperkalemia associated with postsplenectomy thrombocytosis following trauma as well as hepatosplenic schistosomiasis infection.^10^–^11^ Familial pseudohyperkalemia is an autosomal dominant, albeit exceedingly rare, disorder in which erythrocyte plasma membranes exhibit temperature-dependent permeability of potassium in vitro.^12^ Although vastly different pathologies, it can be inferred that processes that promote cell fragility increase the risk of pseudohyperkalemia. Thus, clinical suspicion for pseudohyperkalemia should be maintained in patients with these predispositions. Likewise, underlying hematological disorders should be considered in patients with suspected pseudohyperkalemia without known disease.

**CPC-EM Capsule**

What do we already know about this clinical entity?

Pseudohyperkalemia is an in vitro increase in serum potassium associated with thrombocytosis and leukocytosis, processes that increase cell fragility and lysis.

What makes this presentation of disease reportable?

Reported cases of pseudohyperkalemia, especially relating to unusual causes such as leukemia, contribute to the limited body of knowledge currently in the literature.

What is the major learning point?

Differentiating true hyperkalemia from pseudohyperkalemia is imperative as the inappropriate treatment of pseudohyperkalemia can lead to devastating hypokalemia.

How might this improve emergency medicine practice?

Maintaining suspicion for pseudohyperkalemia in the appropriate clinical setting will decrease the frequency of inappropriate treatment.

Differentiating true hyperkalemia from pseudohyperkalemia is important as hyperkalemia in patients with leukocytosis in the setting of known or suspected malignancy is concerning for tumor lysis syndrome (TLS), an oncologic emergency involving lysis of malignant cells and extracellular release of potassium and phosphate as well as the generation of uric acid. Treatment of hyperkalemia in TLS is similar to that of hyperkalemia in other patients.

The inappropriate treatment of pseudohyperkalemia can lead to devastating hypokalemia. Nevertheless, multiple case reports as early as the 1980s have cited the inappropriate treatment of hyperkalemia in hyperleukocytosis.^6^,^13^ We aim to add to the body of knowledge of pseudohyperkalemia. Inappropriate treatment of pseudohyperkalemia can be detrimental. It is imperative that treatment be started with appropriate clinical suspicion rather than solely laboratory findings, which have inherent limitations and errors.

## CONCLUSION

Differentiating true hyperkalemia, a medical emergency, from pseudohyperkalemia, where treatment can be detrimental is important for the EP. In order to do this, the EP needs to be familiar with the plethora of causes of pseudohyperkalemia from the more common hemolysis to the much rarer thrombosis and extreme leukocytosis or thrombocytosis.
